# The Cytoarchitecture of the Tectal-Related Pallium of Squirrelfish, *Holocentrus* sp.

**DOI:** 10.3389/fnana.2022.819365

**Published:** 2022-04-28

**Authors:** Leo S. Demski, Joel A. Beaver

**Affiliations:** Division of Natural Sciences, Pritzker Marine Biology Research Center, New College of Florida, Sarasota, FL, United States

**Keywords:** vision, brain evolution, telencephalon, optic tectum, teleost, cortex, sensorimotor control, coral reef

## Abstract

The squirrelfish, which live in visually complex coral reefs, have very large eyes and a special dual-system “day and night vision” retina. They also have atypical expansions of brain areas involved in processing visual information. The midbrain tectum sends information *via* diencephalic relay to two enlarged dorsal telencephalic regions. The latter include a superficial dorsal/lateral “cortex-like area” of small to medium-sized cells [area dorsalis telencephali, pars lateralis-dorsal region (dorsal segment); Dld1] which projects to an underlying dorsocentral region of relatively large cells (the area dorsalis telencephali, pars centralis-dorsal region; Dcd) which in turn reconnects with the tectum. Additionally, the cerebellum is also involved in this pathway. The hypertrophied pallial regions, termed the tectal-related pallium (TRP), most likely exert major influences on a variety of visually-related sensorimotor systems. This research aimed at better establishing the cellular structures and possible connections within the TRP. Nissl and rapid Golgi staining, biotinylated dextran amine tracing and cell-filling, and electron microscopy were used in this study. For gross observation of the pallial areas and plotting of the study sites, a mini-atlas of transverse and horizontal sections was constructed. This research better documented the known cellular elements of the TRP and defined two novel cell types. Species differences in the TRP may be related to possible differences in behavior and ecology.

## Introduction

The squirrelfish (SF) are a group of widely distributed coral reef teleosts recognized by their red and white color and large eyes (Randall, 1996; Greenfield, 2002; Nelson et al., 2016; Robertson and Van Tassell, 2019). The fish are primarily territorial-corpuscular or nocturnal predators that feed on crabs and shrimp and occasionally other taxa including mollusks and fish (Hobson, 1974, 1975; Gladfelter and Johnson, 1983). With the soldierfish, the SF form the Holocentridae (Nelson et al., 2016).

Besides the flashlight fish (Anomalopidae), most SF relatives are mesopelagic marine fishes. As such, SF have a specialized deep-sea like-retina of multiple banks of rods but one that uncharacteristically also contains double and single cones. Thus, while they have an excellent scotopic vision, they probably also have a dichromatic cone-based color vision (useful for diurnal activities) and possibly also a rod-bank-based color vision (useful for seeing colors) in dim light (de Busserolles et al., 2021). The findings are consistent with observations that, while SF forage across patch reefs in dim light, they are also very active near their shelters in the day, e.g., taking food when offered and aggressively defending their territories (personal observations; refer to above references).

Squirrelfish are considered an actinopterygian group basal to the advanced perciforms (most teleosts) with which they appear to share many derived brain structures such as a complex pretectum that most likely developed in a common ancestor in relation to the complex visual environment of the developing coral reefs (Wullimann, 1997; Striedter and Northcutt, 2020). The SF and soldierfish are also noted for their highly developed hearing and sonic activities (Carlson and Bass, 2000; Luczkovich et al., 2000; Luczkovich and Keusenkothen, 2007; Parmentier et al., 2011).

Squirrelfish, with their exceptional eyes, have been subjects for multiple studies of teleost vision. Observing blinded and half-blinded SF, Meader (1934) concluded that vision is necessary for feeding. He mapped retinal projections using normal anatomy and fiber degeneration following unilateral enucleation. He also illustrated: expanded-pleated optic nerves; a very thick tectum with an enlarged torus longitudinalis (TL); a highly developed diencephalic nucleus (“nucleus prethalamicus,” nPTh) with no observed retinal input (later confirmed by Campbell and Ebbesson, 1969) but suggested strong ipsilateral tectal connections; and an expanded dorsal telencephalic area that uncharacteristically fused across the midline. Regarding the latter, Aronson (1963) published a photograph of a myelin-stained transverse section through an expanded dorsal “cortex-like” area overlying a large central region of the SF telencephalon that fused across the midline. The regions are known subsequently as the area dorsalis telencephali, pars lateralis-dorsal region (dorsal segment), or Dld1 and the area dorsalis telencephali, pars centralis-dorsal region, or Dcd, respectively. Aronson also illustrated a densely myelinated fiber “capsule” separating the regions. The two nuclear zones have been referred to as the “tectal-related pallium” (TRP; Demski and Beaver, 2001; Demski, 2003, 2013). Brain-size studies in diverse teleosts have verified the relatively large size of the SF tectum/TL (Kishida, 1979; Schroeder et al., 1980) and the telencephalon including the TRP (Bauchet et al., 1977, 1989; Ridet and Bauchot, 1990). Of special note are observations that, while tectal size is usually reduced in nocturnal fishes, this is not the case for SF (Iglesias et al., 2018).

The cytoarchitecture and certain connections of the SF tectum/TL (Campbell and Ebbesson, 1969; Kishida, 1979; Ito et al., 1980a,b; Schroeder et al., 1980; Vanegas and Ito, 1983a,b; Xue et al., 2003a) and the nPTh (Ito and Vanegas, 1983a,b; Xue et al., 2003a,b) have been detailed, including the associations of the structures with the Dld1 and Dcd. An ipsilateral-topographical “loop” from the tectum *via* a relay in nPTh to Dld1 with a return to the tectum and nPTh *via* Dcd efferents has been well established (Ito and Vanegas, 1983a,^1984^; Demski and Beaver, 2001; Demski, 2003, 2013; Xue et al., 2003a,b). In addition, the Dcd may influence tectal-related TL and cerebellar functions and their interactions through a projection to a cell group adjacent to the posterior commissure (nucleus paracommissuralis, nPC) which has connections to the TL and cerebellum. The cerebellum projects to the nPTh, hence possibly forming an additional “loop” within a TRP “system” (Xue et al., 2003a). Tectal-nPTh-dorsal telencephalic pathways also appear in other fish groups (see Hagio et al., 2018).

Detailing the TRP is necessary for understanding tectal/TL and cerebellar relationships to the dorsal telencephalon in SF. Thus, this research aimed at better establishing SF-TRP interconnectional and cytoarchitectural relations. Nissl, Bodian, and rapid Golgi staining, biotinylated dextran amine (BDA) tracing and cell-filling, and electron microscopy (EM) were used in this study. The BDA tract-tracing also confirmed some of the assumed “extrinsic” connections of the SF TRP.

A comprehensive mapping of the SF telencephalon is not available. Thus, a topographical Atlas of SF telencephalic internal anatomy was constructed for accurate location of the sites of the present cytoarchitectural studies and for the use as a database for future investigations. The Atlas is not intended as a comprehensive study in itself, i.e., with detailed comparisons to other species and information such as nuclear sizes and cellular densities.

Following the Atlas, the sections of the Results consider the Dld1 including its projections through the capsule into Dcd, the capsule, including its intrinsic cells, and the Dcd. A number of two new cell types are described, and a Golgi-based summary of the observed components of the TRP is provided.

The present findings are incorporated into a graphical model (refer to “Discussion” section), which focuses on the analysis of the anatomical relationships within the SF TRP. The major extrinsic connections of the region are related to possible functions of the TRP. The studies in other fish groups are considered as they contribute to the interpretations of the present results and shed light on the evolution of the TRP.

## Materials and Methods

### Species

The use of two species of *Holocentrus* with data from each mixed throughout this study was a necessity. Initially, *H. rufus* were caught, (with permission of the Honduran Department of Fisheries) by the staff at the Roatan Institute of Maine Science, Anthony’s Key, Roatan, Honduras (where the study was initiated), and therefore, the work began on this species. As the study progressed, at times, only the closely related *H. ascensionis* were available and it was also incorporated into the project. Later in this study, a supplier (Reef Displays of the Florida Keys Inc., Marathon, FL, United States) sent mostly the combinations of both species back to our laboratory in Sarasota.

The two species are very similar in shape, color, and behavior (Gladfelter and Johnson, 1983; Greenfield, 2002). The brains of the two species are grossly identical. Indeed, no one in our laboratory could differentiate similar sections of the telencephalon of one species vs. the other. Each figure indicates which of the species is represented.

### Fish Maintenance

The fish used in this study in Sarasota were housed in our new Pritzker Marine Biology Research Facility of which LD was the Director and JB was the Facility Manager. Fish waiting for experimental treatment were maintained at room temperature (ca 68–72° Fahrenheit), which is within their normal temperatures on reefs in Honduras (personal observations). They were placed in a rectangular 900-gal fiberglass tank with a number of rocks and other hiding places and its own filtration system. The fish were fed shrimp and cut fish. In our BDA experiments, post-operative animals (about 36 h) were maintained segregated in 40–50-gal marine aquariums fitted with separate filtration systems. Our professional staff of aquarists had the responsibility for the care of the animals under the supervision of JB. As a part of the University of South Florida at the time, our facility was accountable to the University Research Veterinarian and his staff. In addition, we were required to individually be recertified for handling the fish one time a year. The overall well-being of the animals was under the control of the University of South Florida Office of Research, Division of Research Compliance, Institutional Animal Care and Use Committee (IACUC file #1345 and #1740). These provisions were cited in all grants and publications. Our facility underwent two inspections a year. The authors are highly experienced aquarists and fish biologists.

Squirrelfish in Honduras were held in a trough with continuous flowing marine water. In less than 2 days, the animals were deeply anesthetized and perfused for routine Nissl staining or euthanized and their brains paced in Golgi fixative (see below).

### Euthanization

Squirrelfish were sacrificed by deep anesthesia in MS222 (tricaine methanesulfonate, Finquel, Argent Chemical laboratories, Redmond, WA, United States) at a 1:1,000 solution, i.e., until all movement and breathing had ceased. The perfusions of buffer and fixative were performed while the heart was still beating.

### Photography/Drawing, Cell Measurements, and Nomenclature

Some photomicrographs are combined multiple images (montages of several types). This work was done using Adobe Photoshop (Adobe Systems Inc., San Jose, CA, United States). Other photographs and drawings were processed to allow for brightness and contrast errors in the original micrographs or drawings and to colorize three of the figures. In addition, lines and text were added in Adobe Photoshop. Some original errors in these procedures were partly erased and corrected. Absolutely, no changes were made that influence the anatomical (scientific) information of any of the figures.

For cell size approximations (refer to definitions of cell types in “Results” section), over 50 “principal cells,” 50 “bridge cells,” and 75 Dcd neurons were measured. Since our sample size of “completely” isolated “capsule cells” was small, we only measured our four best examples. The longest dimension of the cell body was determined primarily using Nissl and BDA material. These findings were consistent with Golgi and EM results. For the most part, modifications of the nomenclature of Nieuwenhuys (Meek and Nieuwenhuys, 1998) were used in the naming of pallial structures.

### Golgi Staining

This manuscript primarily relies on the results of rapid Golgi studies carried out over a period of years. The method requires “fine tuning” not only for species but also other important factors such as age, brain area, and cell type, and even so, the technique is “finicky” (refer to Scheibel and Scheibel, 1970; Valverde, 1970). Thus, repeated trials with variations of staining procedure were necessary to reveal the four major cell types identified in the TRP. The basic rapid Golgi method of Valverde (1970) with some modifications by Morest and Morest (1966) was used (see Evan et al., 1976). Modifications for SF brains include slight variations of immersion fixation and “staining” times. Tissue was embedded in celloidin and sections were cut between 100 and 150 μm in all three planes using an American Optics sliding microtome (model 860-now under Reichert Inc., Depew NY, United States) (Kiernan, 2015). Using a Wild M20 compound microscope (Heerbrugg, Switzerland), areas of interest were traced using 20–100 magnification eyepieces with a camera lucida or later a video screen. Stained cells were photographed using an Olympus BX40 compound microscope and DP71 camera system (Olympus scientific, Waltham, MA, United States). This Golgi study entailed the use of 38 brains (20 transverse, 8 horizontal, and 10 sagittal series).

### Nissl Staining

Animals were perfused with 0.1% benzocaine, teleost Ringers solution followed by 4% paraformaldehyde. A number of three sets of transverse sections were cut through the telencephalon. Our routine procedure was followed (Humason, 1979), and frozen sections were cut using a Reichert Jung Model 380/381 cryostat 1800 and stained with cresyl violet. For the Atlas, six series of brain sections through the telencephalon were used (two sets in each of the three planes of section). Fish were perfused with teleost Ringers solution followed by 4% paraformaldehyde and brains processed as described for celloidin embedding (Kiernan, 2015). They were stained with cresyl violet and cut at 100 μm on the A.O. sliding microtome.

### Bodian Staining

A number of two sets of serially sectioned brains (one each of *H. rufus* and *H. ascensionis*) were available from a previously unpublished study. Both fish used were obtained at the Institute of Marine Science in Roatan Honduras, by permit of the Honduran Department of Fisheries. Methods were as described in Kiernan (2015).

### Biotinylated Dextran Amine Fills

For this study, BDA (lysine fixable BDA, Molecular Probes, Eugene, Oregon) implants were used to retrogradely fill the cells; in some cases, the procedure resulted in Golgi-like representations. Fish were initially anesthetized in a solution of MS222 at a dilution of 1:10,000 in seawater until they were placed in a holder with aerated seawater with a solution of MS222 at 1:20,000 perfusing their gills. All areas of incision were treated with lidocaine before surgery. A small area of bone was removed from the area above the target brain region of BDA application, and an implant [small fragments of nitrocellulose filter paper (ca 200 μm) dried after being soaked in a concentrated DBA solution] was inserted into a small incision in the meninges. The implant area was covered with surgical foam (Gelfoam Dental Sponge; Pharmacia and Upjohn, Kalamazoo MI, United States) and a small plastic cover glued (cyanoacrylate) on the skull over the implant. Animals were revived by pumping fresh seawater over their gills and returned to their holding tanks. Fish were sacrificed 36–48 h after the surgery and perfused with 0.1% benzocaine, teleost Ringers, and a fixative of 10% sucrose and 4% paraformaldehyde. Brains were placed in the fixative for at least 48 h and embedded in Tissue-Tek OCT compound embedding medium (Ted Pella Inc., Redding, CA, United States). Frozen sections were cut at 40 μm on a Reichert Jung Model 1800 cryostat. Sections were mounted on the gelation coated slides and processed for staining with avidin-biotin-HRP (horseradish peroxidase) and 3,3′diaminobenzidine (DBA) using a Vector Laboratories (Burlingame, CA, United States) Vectastain Elite ABC Kit. BDA implant sites: area restricted to Dld1 just above the filled-fiber bundles in the capsule; small implant in Dld1/capsule within 1 mm of the filled cell; an implant that covered all tectal layers in the ipsilateral rostromedial tectum; a moderate-sized ipsilateral implant near the midline in Dld1 and parts of the capsule and peripheral Dcd. A total of six fish were used in this study.

### Plastic Embedding Electron Microscopy and Light Microscopy

Brains of one each of *H. rufus* and *H. ascensionis* were embedded in plastic for EM and ca. 1-μm thick sections (TS) for light microscopy. Samples were taken in the transverse plane of both the Dld1 and central Dcd. Fish were anesthetized in a solution of MS222 (as above) and perfused through the heart with 0.1% benzocaine followed by 2% paraformaldehyde 2% glutaraldehyde in either 0.1 M phosphate buffer (fish 1) or 0.1 M cacodylate buffer (fish 2). The brains were removed and cut in slabs on a Series 1000 Vibratome (Technical Products International, St. Louis, MO, United States), and the slabs were held in the above fixative(s). All samples were washed in buffer and post-fixed for 1 h in 1% osmium tetroxide in 0.1 M cacodylate (pH 7.4). Samples were dehydrated (10 min each) in a graded series of ethanol solutions then placed in 2:1 L.R. White Resin (Electron Microscopy Sciences, Hatfield, Pennsylvania)/100% alcohol and infiltrated in L.R. White Resin overnight. The tissue was sectioned using a Sorvall MT-2 ultramicrotome (Sorvall Inc., Norwalk, CT, United States). Thin sections stained with uranyl acetate and lead citrate were mounted on the grids using standard methods (Hayat, 2000) and examined with a Phillips CM 10 electron microscope (Phillips Electronics, Eindhoven, Netherlands). TS were stained with toluidine blue and mounted on glass sides and studied using Leitz Orthoplan II (Ernst Leitz Wetzlar GmbH, Wetzlar, Germany) and Olympus photomicroscopes (as described above).

## Results

### Relational Atlas of the Telencephalon

**FIGURE 1:**
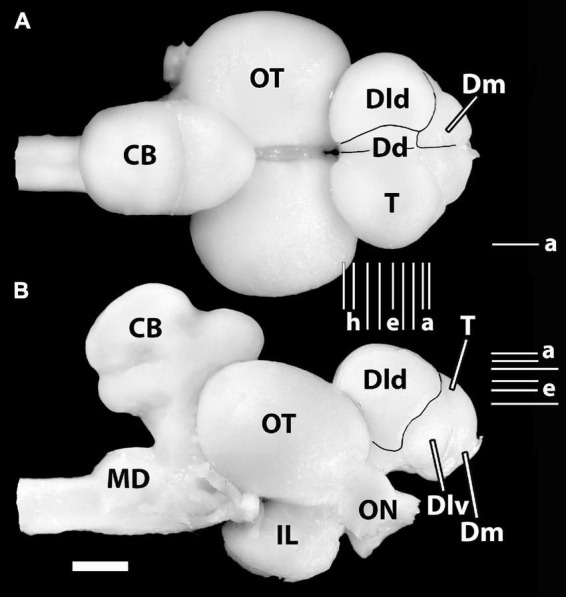
Photographs of the brain of *H. ascensionis* **(A)**, dorsal view, **(B)** lateral view. Major areas of the pallium (Dd, Dld, and Dm) are outlined. Vertical Lines (a–i) between **(A)** and **(B)** represent the estimated levels of the transverse sections in Figure 2. The Horizontal Lines (a–f) in panel **(B)** indicate the estimated levels of the horizontal sections in Figure 3. The horizontal Line (a) in panel **(A)** indicates the estimated level of the sagittal section in Figure 4B. CB, cerebellum; Dd, area dorsalis telencephali, pars dorsalis; Dld, area dorsalis telencephali, pars lateralis-dorsal region; Dlv, area dorsalis telencephali, pars lateralis-ventral region; Dm, area dorsalis telencephali, pars medialis; IL, inferior lobe; MD, medulla; ON, optic nerve; OT, optic tectum; T, telencephalon. Scale bar 2 mm.

Meader’s (1934) drawings of dorsal and lateral views of the SF brain correspond with our photographs in Figures 1A,B. The telencephalon is large and overlaps the rostral part of the tectum. Hypertrophied optic nerves enter an enlarged tectum which overlies a well-developed inferior lobe. The cerebellum is also expanded. Dorsally, a rostral tongue-like protrusion extends over the part of the caudomedial tectum. A sulcus separates this area from a more caudal dorsal lobe which is divided from a ventral lobe by a sulcus. The medulla is unremarkable. The sessile olfactory bulbs are close to mid-rostrocaudal levels of the telencephalon.

**FIGURE 2:**
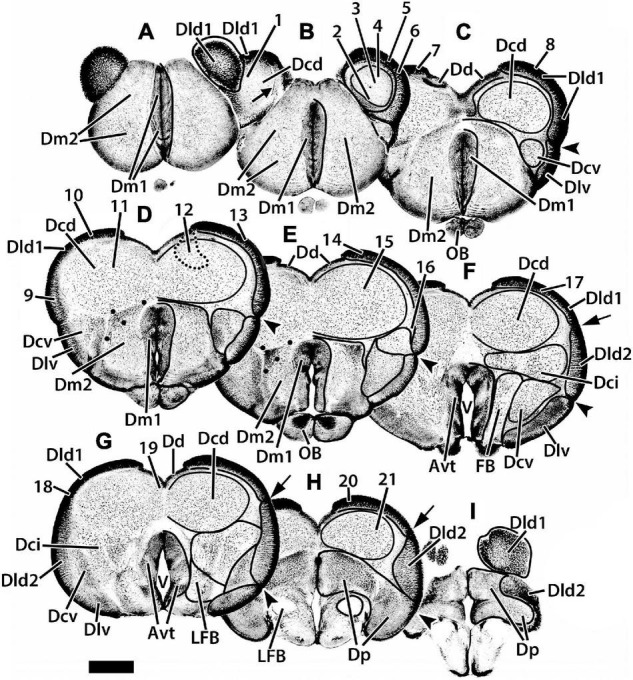
Representative transverse sections **(A–I)** of the telencephalon of *H. rufus.* The estimated levels of the sections are related to the whole telencephalon in Figure 1. Brain areas are indicated by straight lines from marginal abbreviations and “encircled” on the left side of each section to further indicate their extent. Numbers and their connected lines indicate the estimated position of sites in the figures of this manuscript (22 from transverse sections and two transposed from sagittal sections; i.e., sites 7 and 11). The electron micrographs and sites from sectioned thick-plastic material were taken from two positions, site 14 for Dld1 [area dorsalis telencephali, pars lateralis-dorsal region (dorsal segment)]; and site 15 for Dcd (area dorsalis telencephali, pars centralis-dorsal region) on section **(E)**. Site 12 indicates the general area (enclosed by the dotted lines) of several cells. The arrow in section **(B)** marks a blood vessel in the capsule deep to Dld1. The large dots in sections **(D,E)** indicate a specialized region of Dm2 (area dorsalis medialis-lateral nucleus); see text for details. Avt, area ventralis of the telencephalon; Dci, area dorsalis telencephali, pars centralis-intermediate region; Dcv, area dorsalis telencephali, pars centralis-ventral region; Dd, area dorsalis telencephali, pars dorsalis; Dld2, area dorsalis telencephali, pars lateralis-dorsal region (ventral segment); Dlv, area dorsalis telencephali, pars lateralis-ventral region; Dm1, area dorsalis telencephali, pars medialis-periventricular nucleus; Dm2 area dorsalis telencephali, pars medialis-lateral nucleus; Dp, area dorsalis telencephali, pars posterior; LFB, lateral forebrain bundle; OB, olfactory bulb; V, telencephalic ventricle. Scale bar 1 mm.

**FIGURE 3:**
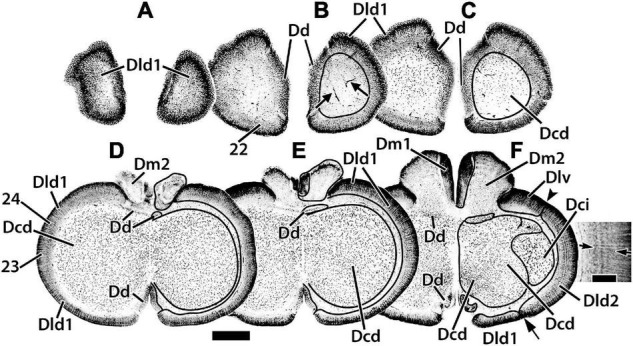
Representative horizontal sections **(A–F)** of the dorsal telencephalon of *H. ascensionis*. The estimated levels of the sections are related to the whole telencephalon in Figure 1. Brain areas are indicated by straight lines from marginal abbreviations and “encircled” on the left side of each section to further indicate their extent. Numbers and their connected lines indicate the estimated position of sites from horizontal sections in the figures of this manuscript (sites 22–24). The arrows in section **(B)** mark blood vessels in the capsule deep to Dld1. Note the horizontal and vertical stratification in Dld2 in section **(F)**. The insert in section **(F)** is a higher magnification of the adjacent part of the main figure. Arrows denote two prominent and a third less well-defined “concentric” layers of cells; radial columns of Dld2 cells are also evident (see text for details). Dcd, area dorsalis telencephali, pars centralis-dorsal region; Dci, area dorsalis telencephali, pars centralis-intermediate region; Dd, area dorsalis telencephali, pars dorsalis; Dld1, area dorsalis telencephali, pars lateralis-dorsal region (dorsal segment); Dld2, area dorsalis telencephali, pars lateralis-dorsal region (ventral segment); Dm1, area dorsalis telencephali, pars medialis-periventricular nucleus; Dm2, area dorsalis telencephali, pars medialis-lateral nucleus. Scale bars main panel 1 mm; insert 200 μm.

In general, six subdivisions or zones of the area dorsalis telencephali (i.e., the pallium) of eutelosts have been generally recognized (Yamane et al., 1996; Meek and Nieuwenhuys, 1998; Northcutt, 2006, 2008, 2011; Yamamoto, 2009; Mueller et al., 2011; Demski, 2013). In our material, four of them form surface structures (Figures 1A,B). The medial region [area dorsalis telencephali, pars medialis (Dm)] appears as an exposed “rostromedial lobe” that continues ventrocaudally as a deep structure. The dorsal part [area dorsalis telencephali, pars dorsalis (Dd)] and the lateral component [area dorsalis telencephali, pars lateralis (Dl), which is divided into dorsal (Dld) and ventral regions (Dlv)] form a “dorsal-lateral lobe” that covers the deeper structures of the “hemisphere.” Note, we cannot define a posterior part of Dl (i.e., a Dlp) as described in some acanthopterygians (Northcutt and Braford, 1980; Northcutt and Davis, 1983; Cerdá-Reverter et al., 2001; Dewan and Tricas, 2014). Deep structures in the SF brain include the following: the posterior region of the pallium [area dorsalis telencephali, pars posterior (Dp)], a ventrocaudal structure, and the central part [area dorsalis telencephali, pars centralis (Dc)] with dorsal (Dcd), intermediate (Dci), and ventral (Dcv) regions. Thus, Dp and Dc are not exposed in Figure 1. All of the pallial regions and the area ventralis of the telencephalon (Avt) are illustrated in the appropriate sectional figures (Figures 2, 3).

We have identified two components of Dm (area dorsalis telencephali, pars medialis). Dm1 (area dorsalis telencephali, pars medialis-periventricular nucleus) and 2 (area dorsalis telencephali, pars medialis-lateral nucleus) form the rostral pole of the telencephalon, i.e., the “rostromedial lobe” (Figures 1A,B, 2A,B, 3D–F). Caudally, they are covered dorsally by the Dcd (Figures 2C–E); both Dm nuclei terminate caudally at the level of the Avt (Figure 2F). Dm1 is a periventricular nucleus associated with medial Dm2. It has densely packed small “granular” cells. Dm2 forms the bulk of the Dm and has medium-sized cells “uniformly” distributed throughout its central zone. The peripheral aspect of Dm2 appears “specialized” with a medial zone of several distinct cellular layers running parallel to the surface and a more lateral region of “uniformly” distributed densely packed cells (Figures 2B–D). At mid-telencephalic levels, Dm2 also contains a compact “wedge-shaped” area of small granular-like cells which are surrounded by dots on Figures 2D,E. More research is required to determine whether any of these Dm2 sub-regions are separate nuclei. Area dorsalis telencephali, pars dorsalis (Dd), is a ribbon-shaped structure that extends from a midline fusion with the contralateral Dd to the medial edge of Dld. It forms an “arch” over medial Dcd, running from the Dm at the rostral level of the olfactory bulbs to terminate caudally at the dorsal edge of Dp. Throughout, it overlies the medial part of the outer surface of the fiber capsule situated on the dorsal aspect of Dcd (Figures 2C–H, 3C–F). The capsule is readily identified by its conspicuous blood vessels (refer to tangential views in Figures 2B, 3B). Dd is composed of densely packed medium-sized cells.

With its attachment to Dd medially, the Dld continues over the lateral surface of the telencephalon to a sulcus separating it from Dlv (Figures 1, 2C–F). A relatively thin dorsal component of Dld and Dld1 is closely associated with Dcd (Figures 2B–H, 3C–F). A thicker ventrolateral component of Dld, herein designated as area dorsalis telencephali, pars lateralis-dorsal region (ventral segment) or Dld2, is superficial to the lateral aspect of Dci (Figures 2F,G, 3F).

Radial columns of cells are evident throughout Dl and specialized “bridge cells” (BC; refer to the section below) are also present and specific to the area (see below). The Dld2 has several bands of concentrically oriented cells (Figure 3F). In the most caudal regions of the telencephalic lobe, Dld2 continues toward the base of the hemisphere, overlying the lateral aspect of Dp (Figure 2I). As a major component of the TRP, the Dld1 is detailed in a separate section.

Area dorsalis telencephali, pars dorsalis-ventral region (Dlv) which overlies the ventral component of Dcv, extends ventromedially from a sulcus separating it from Dld dorsally. It abuts Dm2 ventrally (Figures 2C–E). Caudally, it continues to the ventral aspect of the telencephalon (Figures 2F,G) where it remains for some distance and is replaced near the caudal pole of the telencephalon by Dp (Figure 2H). Dlv exhibits radial cell columns that are less prominent than those in Dld.

In many teleosts, Dc is composed of several more or less distinct large-celled nuclei to which a variety of names have been applied. As mentioned above, we have identified three putative Dc nuclei in SF (Figures 2, 3). In general, some of the Dc components have been related anatomically to their respective overlying “cortex-like” structures (refer to “Discussion” section; Northcutt and Davis, 1983; Northcutt, 2008; Demski, 2013); of particular interest to this study is the relationship of Dld1 and Dcd, i.e., the major components of the TRP.

Situated immediately ventral to the capsule underlying Dd medially and Dld1 dorsolaterally, the Dcd forms the bulk of the “dorsal-lateral lobe” of the telencephalon (Figures 2B–H, 3C–F). Throughout most of its extent, it is continuous across the midline with the widest fusion at mid-rostrocaudal levels (Figures 2D,E, 3D,E). The distribution and size of Dcd cells are fairly homogeneous (Figures 2, 3). As a major component of the TRP, the Dcd is detailed in a separate section.

Area dorsalis telencephali, pars centralis-intermediate region has the largest cells in the dorsal telencephalon (Figures 2E–G, 3F). They resemble Dcd cells in Golgi material (Mahoney, 2001; Demski, 2013). The lateral forebrain bundle (LFB) courses through the part of Dci, somewhat dividing it into medial and lateral subnuclei (Figures 2F,G). Area dorsalis telencephali, pars centralis-ventral region (Dcv) is difficult to define except where it is near Dlv. In some areas, the border between Dci and Dcv is uncertain. Dcv has fairly dense populations of smaller cells adjacent to Dlv and more dispersed larger cells more centrally (Figures 2C–G). Caudally Dcv is replaced by Dp (Figure 2H).

Area dorsalis telencephali, pars posterior (Dp) forms the caudal pole of the dorsal telencephalon (Figures 2H,I) where it is ventral to Dcd medially and Dld2 laterally (Figures 2H,I). It has relatively densely-packed medium-sized cells. Ventromedially, part of Dp is immediately dorsal to the compact LFB (Figures 2H,I). The subpallial ventral telencephalic area (Avt) is composed of a dense nucleus of small cells surrounding the ventricle. The Avt replaces Dm1 caudally (Figures 2E–G).

### Cytoarchitecture Within the Tectal-Related Pallium

The following subsections describe the components of the TRP following their progression from superficial to deep. A Golgi-based summary/illustration of the structures observed in the Dld1/capsule is provided (see below).

#### The Principal Cells

**FIGURE 4:**
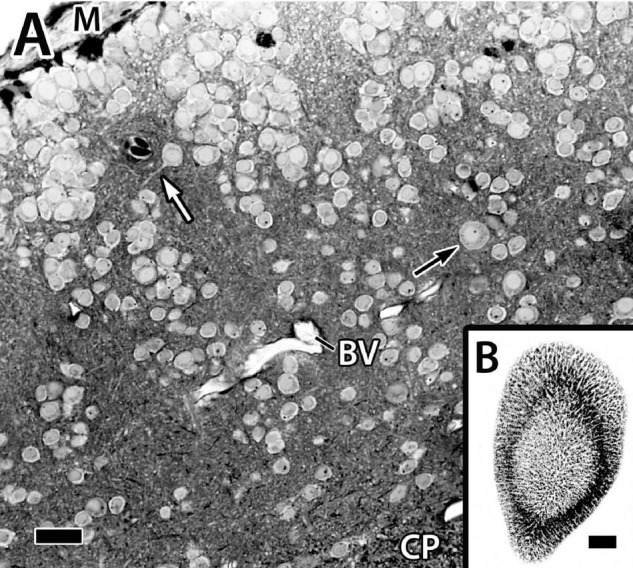
Panel **(A)** is a photomicrograph of a thick plastic section through the Dld1 [area dorsalis telencephali, pars lateralis-dorsal region (dorsal segment)] in *H. rufus* (site 14-Figure 2E). Clusters of principal cells form roughly divided “radial columns” extending from the meninges (M) inward to the fiber capsule (CP). The black arrow indicates a particularly large principal cell and the white arrow marks the dendrite of a medium-sized principal cell. See text for details. Panel **(B)** is a tangential sagittal section showing radial cell columns extending from the surface to just superficial to the CP in *H. rufus*. The estimated level of the section is related to the whole telencephalon in Figure 1. BV, blood vessel. Scale bars **(A)** 1 mm; **(B)** 20 μm.

The gross features of the Dld1 are described in the Atlas. The structure has partially twisted columns of cells that radiate outward from the capsule (between it and Dcd) to the surface. The columns are especially striking when viewed in fairly thick sections of the most rostral, caudal, or lateral aspects of Dld1 where they are seen from the surface “looking inward” to the fiber capsule on which the Dld “sits” (Figures 2A,I, 3A–C, 4B).

Only parts of several columns are seen in the thinner sections because of the twisting (Figure 4A). The radial cell cords are enmeshed in a dense neuropil of myelinated and unmyelinated fibers, small dendrites, and a few scattered blood vessels. With a possible exception (see below), the cells forming the columns appear to be size variations of a similar cell type of small to medium-sized neurons, i.e., ca. 7.5–12.5 μm (Figure 4A, see also below) which we designate as “principal cells” (PC).

**FIGURE 5:**
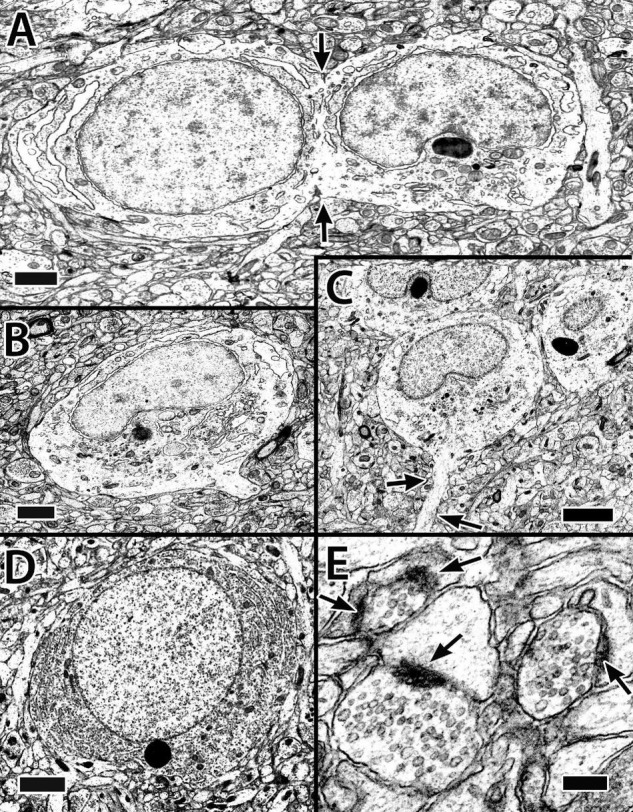
Electron micrographs of principal cells and synapses in the Dld1 [area dorsalis telencephali, pars lateralis-dorsal region (dorsal segment)] in *H. rufus* (site 14; Figure 2E). Principal cells in panels **(A–C)** appear to be similar with a cytoplasm with sparse granular endoplasmic reticulum (ER), an oval to round nucleus with patches of dense chromatin and a concavity associated with the nucleolus. The principal cell in panel **(A)** appears to be dividing (arrows). Apparent dendritic processes are visible in panel **(B)**, lower right and **(C),** arrows. The typical clustering of principal cells is evident in panel **(C)**. The cell in panel **(D)** represents a less common cell-type with a dense granular ER filled cytoplasm and a nearly round nucleus with an even distribution of chromatin. Panel **(E)** demonstrates synapses on dendritic spines in the neuropil surrounding principal cells in Dld1; arrows indicate synaptic thickenings. Scale bars **(A,B)** 1 μm; **(C)** 2 μm **(D)** 1.5 μm; **(E)** 0.2 μm.

A total of two different cytoplasmic and nuclear profiles of cells in the size range and location of principal cells have been identified at the EM level (Figure 5). The “common” type has a round to elliptical soma, an oval nucleus with clumped chromatin and in some views an indentation for a prominent nucleolus. A scant cytoplasm includes some granular endoplasmic reticulum (ER), a Golgi apparatus, a few membranous sacs, and free ribosomes (Figures 5A–C). Some of the cells appear to be dividing or at least sharing cytoplasm (Figure 5A). A “less common” cell type has a nearly round nucleus with uniformly distributed chromatin, a “circular” nucleolus, and dense cytoplasm filled with granular ER (Figure 5D).

**FIGURE 6:**
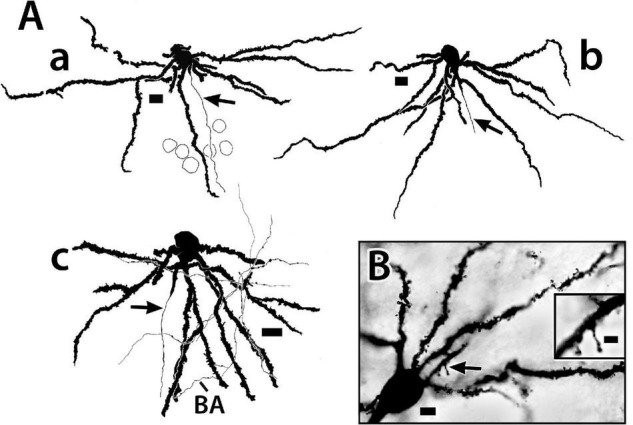
Golgi-stained principal cells from the Dld1 [area dorsalis telencephali, pars lateralis-dorsal region (dorsal segment)] of *H. rufus*. **(A)** camera lucida tracings from a transverse section **(a)** (site 17; Figure 2F), sagittal section **(b)**; (site 7; Figure 2C) and a horizontal section **(c)**; (site 23; Figure 3D). In panel **(A)** the top of the page is toward the surface and the bottom inward toward the fiber capsule. The principal cells have branching-spiny dendrites **(a–c)**. Some dendrites are “cut off” as they exit the plane of section. Two dendrites on the left have missing areas represented by the small gaps. Arrows indicate axons originating from either the central part (lower in figures) of the soma **(a)** or a dendrite in the region **(b,c)**. Beaded axons (BA) appear throughout the Dld1 and closely engage principal cell dendrites as illustrated in panel **(c)**. Outlines of adjacent principal cells are indicated in panel **(a)**. **(B)** photomicrographs of a transverse section. Spines are prominent on the soma-connected dendrites. The arrow marks the region of the insert which shows two spines at higher magnification. See text for details. Scale bars **(Aa–c)** 10 μm; **(B)** main panel 5 μm, insert 2 μm.

The principal cells have dendrites with prominent spines (Figures 6Aa–c,B) and synapse-contacting spines are situated in the neuropil near the cells (Figure 5E). The dendrites generally emanate from all but the most surface-oriented (here defined as superficial) part of the soma. The longest processes (at least up to 125 μm in the larger cells) originate superficial to the remaining dendrites and extend concentrically; i.e., somewhat parallel to the surface. The remaining more centrally positioned (i.e., toward the capsule) dendrites form a wide capsular-directed “fan” (Figures 6Aa–c). Thus, principal cells appear as “hemi-stellate cells.”

**FIGURE 7:**
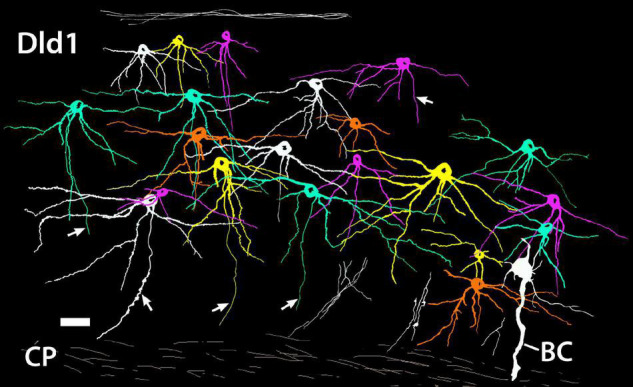
A camera lucida tracing from a *single site* on a Golgi-stained horizontal section (site 24; Figure 3D) through the Dld1 [area dorsalis telencephali, pars lateralis-dorsal region (dorsal segment)] of *H. rufus*. Using Adobe Photoshop, an original hand-colored black and white tracing was “inverted” with virtual coloring added to facilitate appreciation of the 21 individual principal cells. The surface is at the top. A bridge cell (BC) is also present; see definition and descriptions in text. Note the overlapping of principal cell dendrites and the considerable size variation of the principal cells with their dendritic spread at least roughly proportional to soma size. While axons are present on all of the principal cells, for clarity only five were labeled (arrows). Several “unconnected” presumed principal cell fibers are illustrated in the white bundles in the lower right area. Principal cell axons run centrally toward the capsule (CP) where they may branch and develop beads; e.g., the axon approaching the scale bar in the lower left and two of the “unconnected fibers” in the lower right. See text for details. Scale bar, 25 μm.

The relative positions of principal cells within the thickness of the Dld1 can be appreciated from a tracing of twenty-one Golgi-stained cells as they appear *in situ* on a *single site* on a horizontal section (Figure 7). The virtual coloring optimizes visualizing the extent of the individual neurons and their overlap. The figure and our other material (Figure 4A) do not permit a generalization of a specific PC size distribution within Dld1. Dendritic thickness and extent seem directly related to the soma size. Throughout Dld1, the principal cells are contacted by a plexus of fine-beaded axons (Figure 6Ac).

**FIGURE 8:**
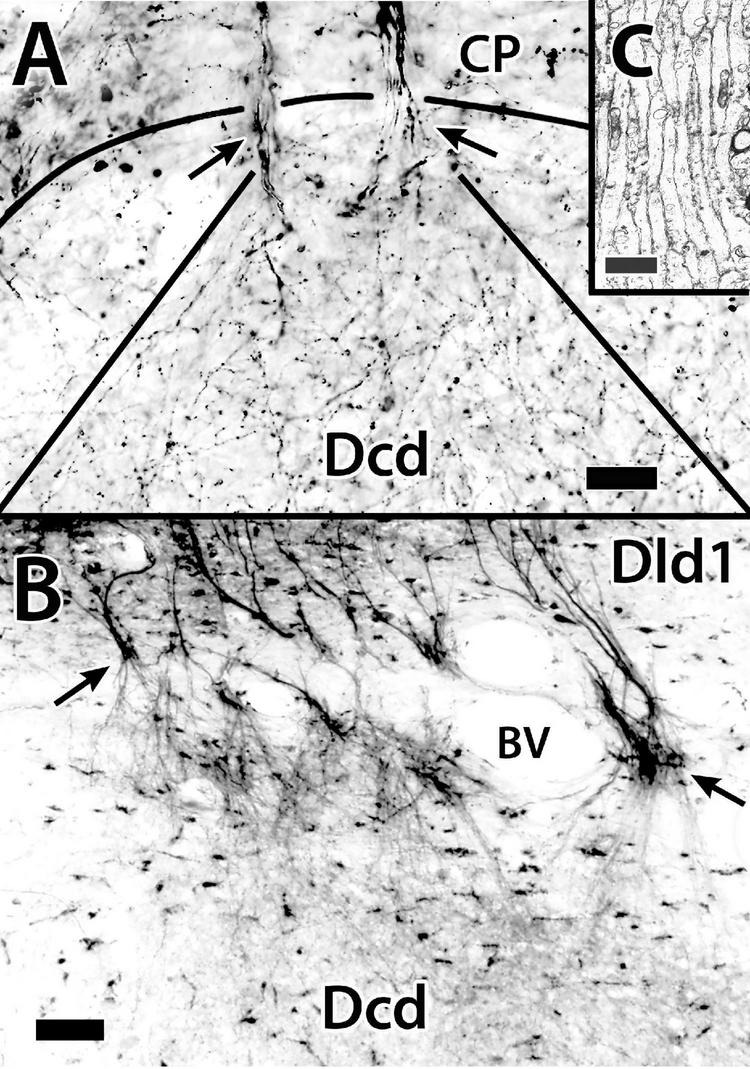
The distribution of principal cell axons in the capsule (CP) and “outer” Dcd (area dorsalis telencephali, pars centralis-dorsal region). Panel **(A)** is a Golgi-stained transverse section in *H. rufus* (site 16; Figure 2E). Two principal cell axon bundles extend through the capsule (CP) into Dcd (arrows) where they branch and diverge forming a plexus of fine-beaded axons. The solid-curved line indicates the capsule/Dcd border. The straight lines mark the general outside boundary of the overlapping cones of distribution of the two bundles. Panel **(B)** is the result of a BDA implant (not shown) into the dorsomedial Dld1 [area dorsalis telencephali, pars lateralis-dorsal region (dorsal segment)], that labels principal cells and their axons on a transverse section in *H. ascensionis* (site 20, Figure 2H). As in panel **(A)**, the labeled fibers form bundles that pass through the capsule (arrows), branch and diverge into a “cloud” of fine-beaded fibers in the subjacent region of Dcd. Panel **(C)** is an electron micrograph of a transverse section through the fiber capsule in *H. rufus* (site 14; Figure 2E). It illustrates a bundle of longitudinally sectioned unmyelinated axons (*ca* 0.3–0.5 μm) presumed from principal cells. See text for details. BDA, biotinylated dextran amine; BV, blood vessel. Scale bars **(A)** 20 μm; **(B)** 50 μm; **(C)** 1 μm.

Unmyelinated axons of principal cells originate from either the basal part of the cell body or a dendrite in the region (Figures 6Aa–c, 7). The fibers run inward toward the capsule where just superficial to, or within the structure, they appear to branch and become beaded (Figure 7). PC axons coalesce into the bundles as they enter and pass through the capsule into Dcd where they break up and spread out into overlapping fans of fine-beaded fibers which may also branch (Figures 8A–C). Collaterals of PC axons have not been observed. Indeed, the fibers are almost straight as they pass from the cells to near the capsule (Figures 6Aa–c, 7).

#### The Capsule and Capsule Cells

**FIGURE 9:**
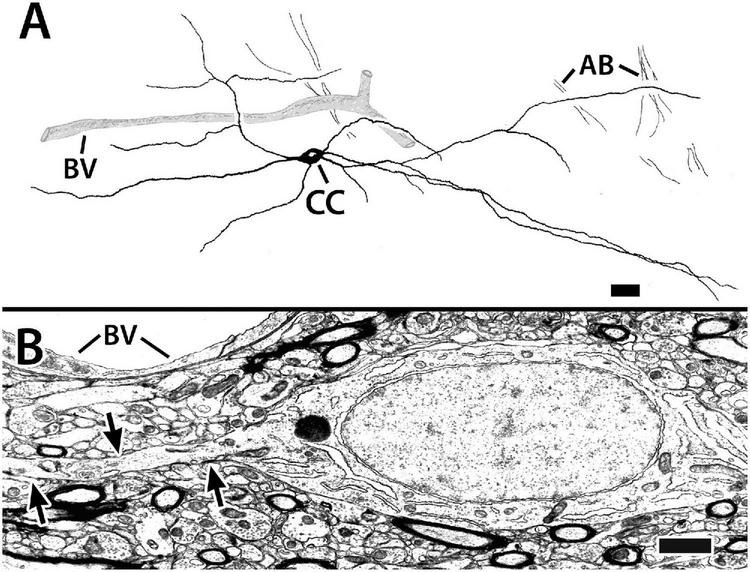
Capsule cells from transverse sections in Dld1 (area dorsalis telencephali, pars lateralis-dorsal region [dorsal segment]) of *H. rufus*. Panel **(A)** is a tracing of a Golgi-stained capsule cell showing its processes (presumed to be dendrites, see text) running “concentrically” in the capsule (site 1; Figure 2B). The capsule cell appears to associate with blood vessels (BV). The dendrites of the pictured cell overlapped those of a neighboring capsule cell (not shown for “simplicity”). Bundles of axons (AB), presumed from principal cells in Dld1, cross “dendritic” fields of the capsule cell. Panel **(B)** represents an electron micrograph of a capsule cell adjacent to a BV (site 14; Figure 2E). The cell’s oval nucleus with dispersed chromatin, a prominent nucleolus and sparse granular endoplasmic reticulum resembles that of the principal cells in Figures 5A–C. The arrows outline one of its processes extending through a dense neuropil with myelinated and unmyelinated processes. Scale bars **(A)** 25 μm; **(B)** 1.0 μm.

The position of the capsule between Dld and Dcd is indicated in the Atlas. As mentioned, it contains bundles of “crossing” PC axons (Figures 8A–C, 9A) which are interspersed within a mass of small dendrites and myelinated and unmyelinated fibers (Figure 9B). The processes of “bridge cells” (BC; refer to definition and figures below) also course across and within the capsule (see below). Many concentrically-oriented blood vessels extend throughout the structure (Figures 2B, 3B, 8B, 9A,B).

The capsule also contains a previously undescribed intrinsic “capsule cell” (CC) which appears bipolar with processes extending from the poles (Figures 9A,B). The processes, which are considered dendrites based on the presence of small spines and their branching patterns, orient concentrically in association with the blood vessels. The dendrites tend to overlap those of other capsule cells and also appear to contact presumed PC axon bundles (Figure 9A). Definitive CC axons have not been identified. In most of our Golgi material, the capsule is too darkly stained to clearly discern CC details.

The somata of capsule cells resemble those of the principal cells in Figures 5A–C. The cell bodies range from 13 to 25 μm in the long dimension with a “thickness “of about 6 μm. The nuclei are elongated in the main axis of the cell. The cytoplasm is sparse with free ribosomes, some ER, and a prominent nucleolus. Chromatin distribution is fairly uniform with some small aggregations (Figure 9B).

#### The Bridge Cells

**FIGURE 10:**
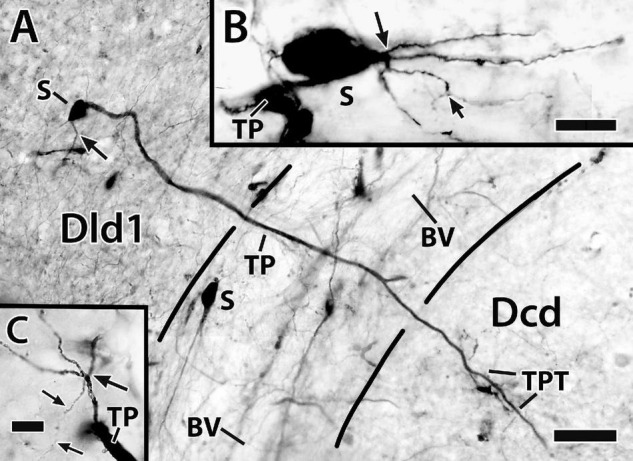
Photomicrographs of bridge cells. In panel **(A)** a BDA implant in Dld1 [area dorsalis telencephali, pars lateralis-dorsal region (dorsal segment)]/capsule labeled a number of nearby bridge cells, two of which are illustrated in this transverse section in *H. ascensionis* (site 13; Figure 2D). One of the cells appears throughout most of its length. Its soma (S) is in the mid-depth of Dld1 and its main thick process (TP) extends across the capsule (area between the dashed lines) where it branches as it approaches Dcd (area dorsalis telencephali, pars centralis-dorsal region). One branch is cut off while the other crosses into Dcd and ramifies into “thick process terminations” (TPT) with brush-like “spines” on and near its endings. See text for details. The arrow indicates an axon-like process (AP) extending from the soma. The second bridge cell, in the outer part of the capsule, is only partially in the section plane. A process with two observable branches extends from the soma (S). **(B)** A bridge cell soma (S) and related structures are represented in a depth-integrated photomontage (nine levels combined) of a Golgi-stained transverse section in *H. rufus* (site 10; Figure 2D). The soma was located just above the capsule in Dld1. A thick process (TP) and three thinner processes (larger arrow) branch from the cell body. The latter extend as “beaded” structures, one of which branches into even finer neurites (smaller arrow). The structures are considered axon-like processes (AP). A tracing of the mostly complete cell is illustrated in Figure 11C. **(C)** A small process extends from a thick process (TP) in a transverse Golgi-stained section in *H. rufus*, (site 18; Figure 2G). The process branches into four neurites which extend various distances. One divides into finer “beaded” processes (smaller arrows). See text for additional details. BV, blood vessel. Scale bars **(A)** 40 μm; **(B)** 20 μm; **(C)** 10 μm.

The “bridge cell” (BC) a “novel” cell type was initially identified in Golgi material of Dld1 and later confirmed as structures filled from BDA implants in the capsule/Dld1 (see below) and observations of cell bodies in Nissl-stained sections of both Dld1 and Dld2. The neurons extend from Dld to Dcd (e.g., refer to Figure 10A) and hence are designated as bridge cells.

**FIGURE 11:**
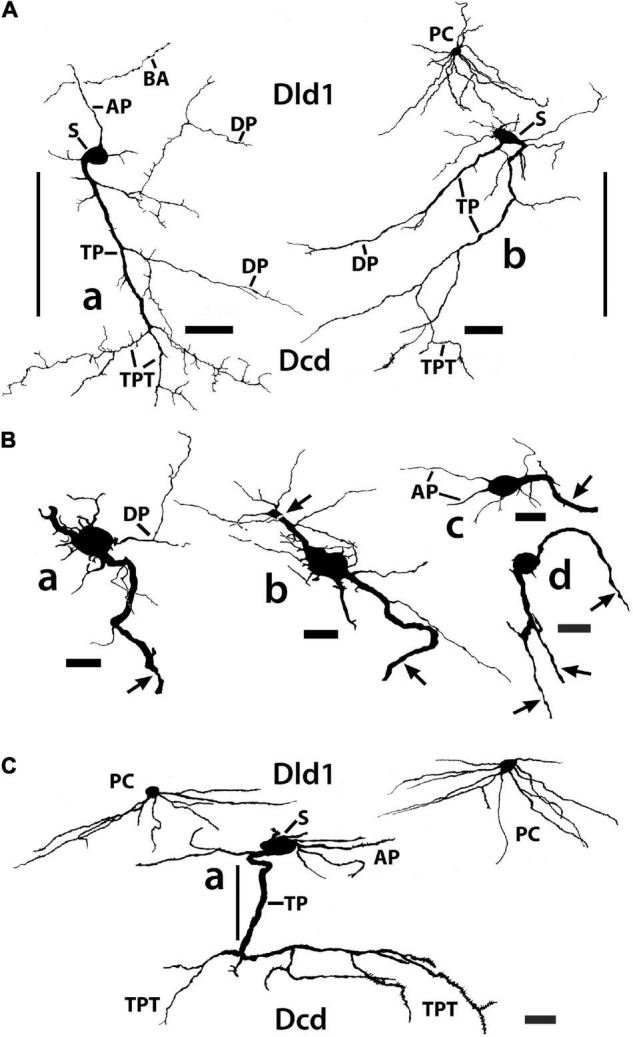
Tracings of Golgi-stained bridge cells and associated structures of Dld1 [area dorsalis telencephali, pars lateralis-dorsal region (dorsal segment)]. The surface is toward the top in the figures. **(A)** Two “mostly” complete bridge cells are from separate transverse sections in the same *H. rufus*: cell **(a)** (site 3; Figure 2B), cell **(b)** (site 8; Figure 2C). The sections were cut tangentially through the capsule between Dld1 and Dcd (area dorsalis telencephali, pars centralis-dorsal region). The vertical bars indicate the general extent of the capsule. A principal cell is included with cell **(b)** for comparison and a beaded axon (BA) crosses a process of the bridge cell in cell **(a)**. One or more thick processes (TP) extend from the cell bodies (S) into the capsule where they branch; some of the thick processes (TP) enter the Dcd where they ramify into “thick” dendritic-like terminations (TPT; thick process terminations) with brush-like processes on the finer endings. Many fine dendrite-like processes (DP), extend from the cell bodies or TP. Some processes appear thickened near the soma and then continue as thinner relatively untapered axon-like processes (AP). **(B)** Cells **(a–d)** are tracings of Golgi-stained bridge cell somata and associated processes in the Dld1 in *H. rufus*. Cells **(a–c)** are from a single transverse section; they represent sites 6, 4, 5 in Figure 2B, respectively. Bridge cell **(d)** is from a horizontal section (site 22; Figure 3B). The arrows in all figures indicate “cut off” branches of thick processes that direct toward the capsule. The upper arrow in cell **(b)** indicates a process “severed” just after recurving toward the capsule. Multiple processes emanate from the cell bodies and to a lesser extent from the TP. Many resemble DP; labeled only in cell **(a)**. Others, practically untapered and often unbranched in the plane of the section, resemble axons [AP in cell **(c)**]. See text for further details. **(C)** Bridge cell **(a)**, on this transverse section of *H. rufus*, is a tracing of the cell from which the photograph of the bridge cell soma illustrated in Figure 10B was taken (site 10; Figure 2D). Two principal cells are shown for reference. The vertical line represents the area of the capsule. The thick process (TP) directly “crosses” the capsule to branch repeatedly in Dcd (modified from Demski and Beaver, 2001, Figure 10, Wiley with permission). Scale bars **(Aa,b)** 50 μm; **(Ba–f)** 20 μm; **(C)** 25 μm.

Bridge cell somata are located in Dld1 usually near the capsule or in the capsule itself (Figures 7, 10A,B, 11A–C). A number of eleven bridge cell somata in a 1.5 mm area along the capsule and the adjacent Dld1 were filled following application of BDA to the adjacent Dld1/capsule area. A number of seven were fairly “evenly” distributed at the same approximate level in the deeper part of Dld, and the others were “scattered” at different levels within the capsule. Such a distribution was confirmed in Nissl-stained sections.

In such material, BC somata range from 16 to 20 μm with a few especially elongate cells approaching 30 μm (Figures 7, 10A,B, 11A–C). In an electron micrograph of an intensely darkened BDA-filled bridge cell, we were able to measure its cell body as 20.6 μm × 10.9 μm with a nucleolus of ca. 4.6 μm; its cytoplasm appeared dense with structural details mostly obscured.

Thin “dendrite-like” processes extend from the bridge cell soma and branch in the Dld1 neuropil while “axon-like” processes have thickened proximal segments that extend from the cell body and remain in Dld1 as fairly straight processes which may have beads and fine branches (Figures 7, 10A,B, 11A–C).

The soma also provides one or more trunk-like “thick processes” which may branch in Dld before extending into the capsule where they can also divide (Figures 7, 10A–C, 11A–C). The thick processes either extend more-or-less “directly” across the capsule to enter Dcd (Figures 7, 10A, 11A,C) or run some distance in the capsule (usually *ca* 100–150 μm, but measured up to 500 μm) before doing so. The structures, which range in diameter from *ca.* 2.5–4 μm with swellings extending over 7 μm, can extend in length at least up to 600 μm (see below). Thick processes taper and end in Dcd as “thick process terminations” (TPT) with fine lateral extensions (Figures 10A, 11A,C).

Dendrite-like processes and perhaps axon-like processes also originate from thick processes within the capsule (Figures 10C, 11A,B). Such dendrite-like processes may remain within the capsule, turn back into Dld1, or enter Dcd (Figure 11A).

#### Summary: Area Dorsalis Telencephali, Pars Lateralis-Dorsal Region (Dorsal Segment)/Capsule

**FIGURE 12:**
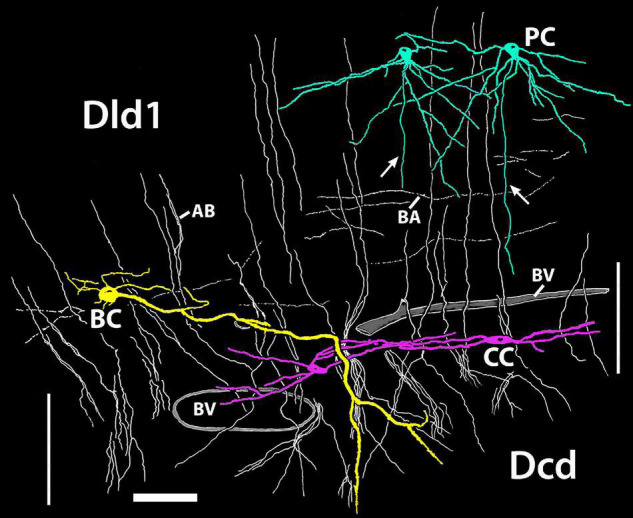
A camera lucida tracing from a single Golgi-stained transverse section in Dld1 [area dorsalis telencephali, pars lateralis-dorsal region (dorsal segment)] of *H. rufus* (site 9; Figure 2D). An original hand-colored tracing was “inverted” with virtual coloring added. The section provides a summary of the observed cytoarchitectonic features of the upper components of the “tectal-related pallium”. The section field extends from the middle to lower part of Dld1 through the fiber capsule (general region extending between the vertical bars on each side of the figure) into the outer edge of Dcd (area dorsalis telencephali, pars centralis-dorsal region). Two principal cells (PC; green) in the upper right have unbranched axons (arrows) directed toward the capsule. Other presumed principal cell axons, some in axon bundles (AB), are indicated in white. Fine-beaded axons (BA) appear to be present throughout Dld1. The soma of a bridge cell (BC; yellow), with extending “small” processes, is slightly dorsal to the capsule on the left side of the figure. It projects a thick process (TP) with a few side branches that are mostly cut-off in the figure. The TP runs across the capsule to bifurcate as it turns into Dcd. Many short fine processes emanate from the BC branches in Dcd. Two overlapping capsule cells (CC; magenta) are closely associated with blood vessels (BV). The TP of the bridge cell overlaps the capsule cell dendrites and descending principal cell axons overlap both structures. Scale bar 50 μm.

Figure 12 is a tracing of a *single site* on a Golgi-stained transverse section (site 9, Figure 2D) that exhibits the three neuronal cell types observed in the Dld1/capsule (highlighted with different colors). As such, it provides a “unique” summary based on an *in situ* condition as opposed to a set of “representative components” (refer to details in the figure legend). Note, bridge cell processes overlap most of the other cellular constituents of the TRP, i.e., beaded axons in Dld1, PC dendrites and/or axons in the Dld1, PC axons in the capsule and Dcd, CC processes, and Dcd dendrites (Figures 7, 11Aa, 12).

#### Area Dorsalis Telencephali, Pars Centralis-Dorsal Region

**FIGURE 13:**
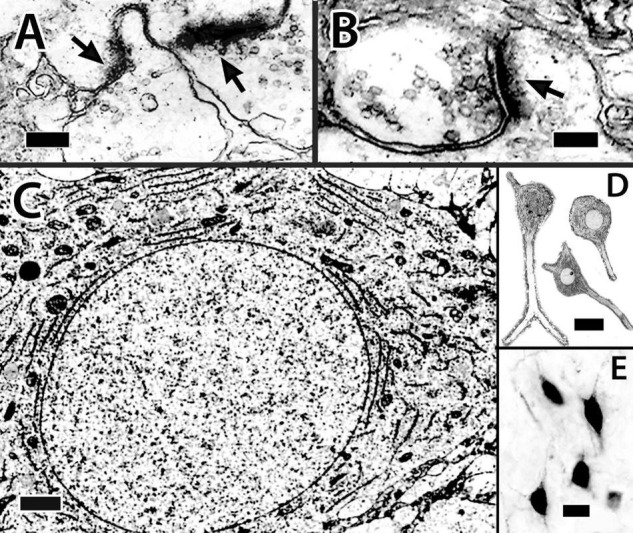
Dcd (area dorsalis telencephali, pars centralis-dorsal region) cells and their processes. **(A,B)** Synapses on spines of a single Dcd dendrite show synaptic thickenings (arrows) and “clear” synaptic vesicles in an electron micrograph in *H. rufus* (site 15; Figure 2E). An electron micrograph of a Dcd soma **(C)** in *H. ascensionis* demonstrates a dense cytoplasm with conspicuous aggregations of granular endoplasmic reticulum, numerous mitochondria and an almost circular nucleus with uniform chromatin (site 15; Figure 2E). **(D)** Dcd cells “cut-out” from a single transverse Nissl-stained thick plastic section in *H. rufus* (site 15; Figure 2E) exhibit a cytoplasmic-dense triangular to “pear-shaped” soma with a round nucleus and a prominent nucleolus (middle cell). Thick dendrites are also evident. A transverse section in *H. rufus* **(E)** shows Dcd cells (site 20; Figure 2H) retrogradely labeled from an implant of BDA into the optic tectum. The labeled cells have soma shapes similar to those in panel **(D)**. See further details in text. BDA, biotinylated dextran amine. Scale bars **(A)** 0.25 μm; **(B)** 0.15 μm; **(C)** 1 mm; **(D)** 20 μm; **(E)** 20 μm.

The following cytological profile of Dcd cells is based on the analysis of: celloidin sections of the Atlas; Golgi material; BDA fills following implants in the tectum, Dld1/peripheral Dcd; 1-μm Nissl-stained plastic sections (TS); and EM. The gross features of the Dcd are described in the Atlas (Figures 2, 3). The areas of Dcd sampled are regions where cells were retrogradely filled following BDA implants in the tectum (Demski and Beaver, 1997; also see Figure 13E).

**FIGURE 14:**
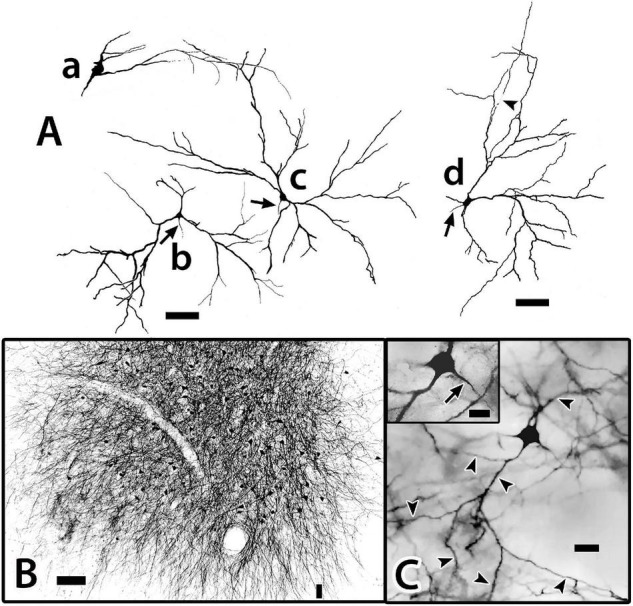
**(A)** The surface is toward the top in all figures. Golgi-stained Dcd (area dorsalis telencephali, pars centralis-dorsal region) cells **(b,c)** appear together with an associated bridge cell **(a)** on an *single transverse section* in *H. rufus*. The estimated area of the three cells is outlined by the dotted lines around site 12 (Figure 3D). The arrows indicate axons **(b–d)**. The bridge cell **(a)** is in the edge of the capsule near Dcd with one of its thick processes extending into Dcd where it overlaps one of the peripheral dendrites of a Dcd cell **(c)**. The pictured Dcd dendrites do not convey their full extent since some were “cut off” because of their great length relative to the section thickness. Cell **(d)** is from a sagittal section (site 11; Figure 2D) which shows a fairly complete set of major dendrites only on the right side. The arrowhead points to a broken portion of a long dendritic segment. **(B)** This transverse section of *H. ascensionis* illustrates the distribution of labeled cells following a small implant of BDA into the area of Dcd adjacent to the capsule near midline (small vertical bar). The figure represents an area of about 1.15 sq. μm (site 19; Figure 2G). At least most Dcd cells and their processes appear to have cell bodies and dendrites extensively labeled resulting in a “web-like” entanglement. Dendritic length can be somewhat appreciated by the dendritic extensions at the periphery of the area of labeled cell bodies. Some of the processes extend across the midline. **(C)** The cell body and several major dendrites (outlined by the arrow heads) of a Golgi-stained Dcd cell are represented in the main figure. This site, which is in an oblique section near the center of Dcd, is not plotted in Figures 2, 3. The insert is a higher magnification view of the cell’s soma and axon (arrow). BDA, biotinylated dextran amine. Scale bars **(Aa–d)** 100 μm; **(B)** 100 μm; **(C)** main figure 25 μm; insert 20 μm.

The “typical” Dcd cell is “polymorphic multipolar.” Some appear triangular or pyramidal-like with three major dendrites originating from the “corners” of the cell body while others are more oval (Figures 13C–E, 14Ab–d,C). Soma size ranges from 22 to 25 μm (longest dimension). The neurons have a round to oval nucleus of 10–12.5 μm with fairly evenly distributed chromatin (Figures 13C,D). A prominent nucleolus is present (Figure 13D, middle cell). The cytoplasm, which also extends into proximal dendrites, is filled with granular ER and mitochondria (Figure 13D).

Satellite cells were closely associated with Dcd cell bodies in some EM samples (not illustrated). A “typical” satellite soma measured 9 μm × 5.6 μm with a nucleus of 3.7 μm × 2.4 μm. The cytoplasm is dense and filled with dark-staining inclusion bodies. The polymorphic nucleus has some dispersed chromatin but also prominent aggregations, especially along the nuclear membrane. The satellite cells resemble perineuronal microglia (Peters et al., 1976; refer to further in “Discussion” section).

Area dorsalis telencephali, pars centralis-dorsal region (Dcd) cells have a few (*ca* 2–4) thick dendrites typically extending over 400 μm (650 μm in Figure 14Ad) from the soma. The main processes “break up” into extended dendritic trees (Figures 14Ab–d,C). The dendrites of many individual Dcd cells overlap, forming a dense “web-like” entanglement which, in addition to blood vessels, essentially “fills” the intersomal space. Some of the Dcd dendrites extend across the midline fusion (Figure 14B). The dendrites have prominent spines that receive synaptic endings (Figures 13A,B). The axons originate from the cell body (Figures 14Ab–d,C-insert). Dcd fills from BDA implants in the tectum indicate that many Dcd axons enter the LFB. The cells form spheres of wide radius with the outer zones providing most of the dendritic surface (Figures 14Ab–d,C). Dcd cells are enmeshed in a fine plexus of beaded axons. At least near the capsule, many such fibers are presumed to be PC axons (Figures 8A,B); the origin of others is unknown. Dcd dendrites also contact branching processes of bridge cells (Figures 14Aa,c).

As a primary part of this study, BDA implants in the tectum, Dld1, and Dcd were used to fill the cells for cytoarchitectural analysis. However, the implants secondarily verified previously published connections of the “TRP extended system.” These include ipsilateral Dcd afferents to the tectum (several layers), nPTh and nPC, and tectal projections to the ipsilateral nPTh (refer to “Discussion” section).

## Discussion

### Atlas

#### The Naming of Area Dorsalis Telencephali, Pars Dorsalis

The identification of Dd as we define it in SF is problematic. The structure’s close relationship with Dl or Dld is key to its identification (Figures 1A, 2, 3B–F). Throughout its extent, the lateral aspect of Dd is closely associated with Dl in gars (Northcutt and Braford, 1980; Northcutt and Davis, 1983) and Dld in certain actinopterygians (Murakami et al., 1983; Yamane et al., 1996; Cerdá-Reverter et al., 2001; Northcutt, 2006; Giassi et al., 2012; Demski, 2013; Dewan and Tricas, 2014). It is also noteworthy that in sturgeons (Northcutt and Braford, 1980; Huesa et al., 2006) and salmonids, Dd and Dld are presented as a single structure (Northcutt and Braford, 1980; Northcutt and Davis, 1983; Folgueira et al., 2004).

The difficulty is that Dd also appears as a “ribbon-like” dorsomedial extension of Dm that arches over much of the medial aspect of the dorsal Dcd (Figures 1A, 2C–H). Thus, as other authors (Ito et al., 1980a,^1982^; Xue et al., 2003a) have done (see below), we initially identified the structure as the part of Dm (Dms in Demski and Beaver, 2001). After consulting with R. G. Northcutt and others, Dd was chosen as the most likely identification of the structure.

#### Components of Area Dorsalis Telencephali, Pars Medialis

Although not the part of the TRP, the area dorsalis telencephali, pars medialis (Dm), nevertheless, deserves special attention. In a variety of teleosts, Dm has strong connections to the Dl/Dld (Murakami et al., 1983; Northcutt, 2006; Giassi et al., 2012; Demski, 2013). Therefore, Dm functions are probably closely associated with those of TRP, and thus, Dm structures are provisionally identified and described.

Area dorsalis telencephali, pars medialis is a complex structure in SF and other advanced acanthopterygians (Cerdá-Reverter et al., 2001; Burmeister et al., 2009; Dewan and Tricas, 2014). Herein, the Dm components are the small-celled periventricular Dm1 and larger more lateral area Dm2. Both areas are common to many fishes (Northcutt and Braford, 1980; Northcutt and Davis, 1983; Ou and Yamamoto, 2016). In SF, Dm2 has three specializations (refer to details in “Results” section) which additional research may reveal as separate nuclei.

### Tectal-Related Pallium: Cytoarchitecture

#### Principal Cells

##### Cytoarchitectural/Functional Considerations

The Dld1 appears as a “thick band” of principal cells that form radial columns spanning the structure from superficial to deep. The principal cells are spiny “hemi-stellate” cells with dendrites on their deeper hemisphere (toward the capsule). Lateral dendrites are the longest and remain fairly straight, thus resembling those of horizontal cells. The other dendrites complete a near 180° fan directed toward the capsule. Principal cells range from small to medium size. Some of the smallest appear to lack the extensive lateral dendrites. The observation suggests a possible second cell type. Gross differences between the larger principal cells were not observed in our Golgi material. In the EM study (with the exception below), most of the principal cells have a rather sparse cytoplasm and a distorted nucleus accommodating a nucleolus; however, in a cluster of such cells, a cell with a round nucleus and cytoplasm filled with Nissl substance was observed. It is one of the larger principal cells (Figure 5D). Thus, the ultrastructural findings also suggest the possibility of two different principal cells. A distribution by cell size in a column was expected, but was not observed (Figures 4A, 7).

Several principal cells (“common type”) appeared to be dividing (Figure 5A). The tight aggregation of similar principal cells is consistent with a grouping of clones. Adult neurogenesis in the pallium occurs in other teleosts (Ganz and Brand, 2016).

A nPTh input to Dld1 is well-established (Ito and Vanegas, 1983b; Xue et al., 2003a; Demski, 2013), and there is evidence that this connection is topographical and hence probably retinotopic (Demski and Beaver, 1997; Xue et al., 2003a). Observations in this study of well-developed PC dendritic spines, Type 1 (symmetrical) synapses on spines near principal cells, and PC contact with beaded axons are consistent with the likely possibility that PC spines receive excitatory nPTh input.

Principal cells probably receive other inputs. In preliminary studies, BDA implanted in Dd (and possibly a part of the underlying capsule) produced a dense fine-fiber plexus in Dld1, suggesting a possible strong Dd input to Dld1 (Demski et al., 1998). There is also preliminary evidence that Dci projects to Dl (Demski and Beaver, 1997) and that Dld1 contains TH-(ir; immunoreactive) fibers and a dense-plexus of fine 5HT-ir axons (Herbsman et al., 2000). Information on other afferents to the SF Dld1 is not available (refer to comments below for results in other fishes).

As observed, PC efferents “blanket” Dcd cell dendrites. This appears to be a strong divergent PC output with the axons branching repeatedly as they approach their final targets. How many Dcd cells a particular size of principal cell affects is not known (see below). PC axons do not appear to synapse with any other cells, as they show no collaterals (or specializations) while passing almost directly through Dld1 to enter the capsule as unmyelinated axon bundles that “break up” in Dcd (Figures 8A,B).

The larger the PC soma, the greater the cell’s apparent dendritic field (Figure 7). Regarding vision, the Dld1 of SF responds electrically to light flashes (Demski, 2003). The bigger cells probably receive greater “retinotopic” input from nPTh. Therefore, the largest principal cells would likely have the widest visual fields. They would also likely have the greatest influence on Dcd cells on which they synapse (see below). This could include a stronger effect on any single cell and/or a distribution to a wider field of cells. Such would most likely affect visual field-related processing in the tectum and possibly the cerebellum *via* the Dcd outputs to these structures (see below). The overlap of the PC dendrites suggests a “smooth” integrative mapping process as would be useful in tracking a moving target.

Regarding the function of the cell columns, the original supposition was that they represent “specialized higher level” information processing units as is typical of laminar structures (Striedter, 2005). With one exception (see “Bridge Cell” below), this idea seems less likely based on the present results. The apparent lack of diversity of Dld1 cell types and PC axonal associations within Dld1 does not seem to provide the necessary “hardware” needed for such functions. More likely, the principal cells may perform “simple integration” of topographically-organized tectal information with inputs from other sources, the output of which may be “amplified” *via* the divergent connections with Dcd cells.

##### Comparative Anatomical Considerations

Golgi-stained cells with spiny dendrites and general configurations similar to principal cells have been observed in the Dl in several Ostariophysians (Sheldon, 1912; Bannister, 1973; Ito, 1973; Schroeder, 1980). The cells are only near the peripheral part of the lobe where their horizontal dendrites run parallel to the meningeal surface and their other dendrites direct inward. Deeper cells in these species have more varied structures being mostly multipolar stellate varieties.

Golgi-stained spiny cells that closely resemble principal cells are in a similar peripheral position in the diencephalic or midbrain inferior lobe in goldfish (*Carassius auratus*) and the bluegill (*Lepomis macrochirus*). The cells have lateral dendrites running parallel to the surface and others directed inward (Demski et al., 1975). Thus, the principal cells may represent a relatively “primitive” non-migratory cell type of the near-surface region in lobular structures. Indeed, the “pyramidal cell” or main output cell in the pallium of the primitive lamprey resembles this cell type with its soma located near the periventricular layer and dendrites fanning out into more superficial fiber layers (Suryanarayana et al., 2021). In advanced acanthopterygians, it appears that this “cell-type” (e.g., the PC) may have proliferated to form the cell columns of Dld1. The adult neurogenesis observed in some principal cells may be a related factor.

The knowledge of Dl/Dld afferents in other teleosts is more advanced than that of SF. The area(s) variously receive input from Dd, Dlv, Dm, the contralateral Dcd, several ventral telencephalic areas (possibly relaying thalamic inputs), locus ceruleus, and the raphé nuclei (Murakami et al., 1983; Yamane et al., 1996; Northcutt, 2006; Yamamoto and Ito, 2008). In addition, the nPTh, which projects to a complex Dld in the yellow-fin goby (*Acanthogobius flavimanus*) (see below), receives afferents from retinorecipient tectal cells (a topographical projection), the retina, ventromedial thalamic nucleus, caudal cerebellum, Dm, and the medial Dc. These results are consistent with Dld being a “visual analysis center” that also functions in multivariate integration.

Like SF, the Dl/Dld1 of other teleosts is filled with TH-ir and 5HT-ir fibers (Hornby and Piekut, 1990; Batten et al., 1993; Kapsimali et al., 2000; Kaslin and Panula, 2001; Vetillard et al., 2002; Lillesaar, 2011).

Area dorsalis telencephali, pars lateralis-dorsal region projects to Dcd in a variety of teleosts (Murakami et al., 1983; Imura et al., 2003; Northcutt, 2006; Yamamoto and Ito, 2008; Giassi et al., 2012). Dl output cells in certain gymnotiform electric fishes are glutamatergic (Giassi et al., 2012). This could be a common condition. Like SF, most of the euteleosts studied have radial columns or “cords” of cells running perpendicular to the Dld surface (Northcutt and Braford, 1980; Northcutt and Davis, 1983; Yamane et al., 1996; Cerdá-Reverter et al., 2001; Muñoz-Cueto et al., 2001; Burmeister et al., 2009). Compared to SF, these fishes, with the exception of the butterflyfish (Dewan and Tricas, 2014; see below), have considerably fewer cell columns, and the columns are wider with more inter-column space. In addition, they form only about a third to half of the superficial to the deep extent of the Dld with the areas deep to columns exhibiting a variety of scattered cells (Yamane et al., 1996). Thus, it appears that SF have thickened and increased the number of columns and compressed or perhaps “lost” the areas deep to the columns.

The Dld in the ocean surgeonfish, the closely-related blue tang, Acanthuridae (Demski, unpublished), and the multiband butterflyfish, Chaetodontidae (Dewan and Tricas, 2014), are grossly similar to the SF Dld1. The four species have some other features in common; i.e., they are visually oriented coral reef fishes that navigate large areas of a complex environment, and they each have a greatly expanded Dcd “covered” superficially with an enlarged area of Dld. Thus, a common environmental constraint may be responsible for the hypertrophy of the TRP in these species.

In terms of the cell type(s) in the columns, only one Golgi study is available for comparison to the present results in SF. Yamane et al. (1996) used the Golgi-Cox technique in the *Sebastiscus marmoratus* and presented drawings of what are incomplete cells (no axons). While no direct relationship to their sites of origin is provided, some of the cells are said to be common in the columns; i.e., small cells and certain larger horizontal cells of the size range of principal cells. However, no single representation compares directly to the PC of SF. Some of their “small” cells are the closest, being similar to the small principal cells of this study.

Two examples of “special” types of Dl need attention as they likely show features that relate to phylogenic and/or ecological diversity. Studies in the yellowfin goby *Acanthogobius flavimanus* (Hagio et al., 2018, 2021) demonstrate an exceptional configuration of a Dl with 17 components. Many of the areas receive afferents from the nPTh but two regions Dl16 and Dl17 have an especially strong input from the structure (Hagio et al., 2021). Conspicuous vertical columns are not reported in any Dl areas. The functions of this Dl are not yet known but gobies have a remarkable distribution as small benthic fishes (Nelson et al., 2016) that live in a variety of aquatic environments including coral reefs. The unique Dl may be a major adaptation to this mode of life.

Even in the “grossly” uniform Dl of the weakly electric fish, *Apteronotus leptorhynchus*, Trinh et al. (2016) have discovered a highly organized pattern of units of Dl composed of “cryptic” layers and columns. The systems appear to be involved in learning, pattern separation, and memory storage (Giassi et al., 2012; Trinh et al., 2016).

#### The Bridge Cells

Bridge cells, a novel cell type, were discovered in the Golgi material (see below). They were later detected in Nissl sections in both Dld1 and Dld2 by their position (near the capsule) and a difference in their cell bodies vs. the other cell types (i.e., more oval with darker cytoplasm). A survey of Nissl-stained sections of Dl in a variety of teleosts revealed cell bodies resembling the “SF-like bridge cells” only in ocean surgeonfish and blue tangs which, as mentioned above, have an “SF-like Dld”. While each bridge cell appears somewhat unique in gross structure, they all appear to have the same general components which are categorized below.

Bridge cells are considered neurons because of the observations of fairly long or thin branching processes that originate from the soma that either resemble axons (some with beads) or look more like dendrites (spines were not apparent). In addition, the latter may originate from large trunk-like thick processes that also emanate from the cell body. The thick processes terminate in Dcd as tapered segments with lateral extensions that resemble long or fine spines.

Several suggestions can be made of possible bridge cell synaptic relations with other cells of the TRP. Afferents to the dendrite-like processes of bridge cells in Dld1 could include any or all of the inputs that the principal cells receive (see above). Functionally, a nPTh-BC connection might be especially important (see below). Bridge cells could also receive unique inputs. PC afferents seem doubtful as no PC collaterals were observed. Axon-like processes of bridge cells most likely target PC dendrites. A BC to BC connection is also possible. Thick processes extend perpendicularly through and horizontally within the capsule. If they are indeed dendritic, they could receive input from any number of myelinated and unmyelinated fibers in the capsule (sources unknown) including possible capsule cell axons (see below). They may contact PC axons but the latter stay in compact bundles with no branches or structures suggesting en passant synapses. The bridge cell thick process terminations overlap Dcd dendrites and the plexus of fine-beaded PC axons that cover the Dcd dendrites. Thus, PC axons may synapse with these assumed dendritic endings of bridge cells. The thick process terminations could also receive other afferents to Dcd or perhaps input from the Dcd cells. A possible functional role of the bridge cell is discussed below.

#### The Capsule and Capsule Cells

A capsule of fibers is associated with the SF Dcd (this study; see also Xue et al., 2003a,b). It is also apparent in fiber-stained studies of other euteleosts (Murakami et al., 1983; Yamane et al., 1996; Mahoney, 2001; Pepels et al., 2002; Demski, 2013).

The capsule contains many fibers of the LFB, or ascending afferents to Dl and Dm and efferents to lower brain areas including the tectum (Meek and Nieuwenhuys, 1998). Where the Dcd has expanded greatly, the capsule is specially enlarged (see Figures 2, 3; Dewan and Tricas, 2014).

The presence of cells within the capsule of SF could be unique. The capsule cells (CC) are bipolar with spiny dendrites branching from either “end” of the soma. Their ultrastructure resembles the “common” type of PC, suggesting a possible similar developmental origin before the capsule fibers enter the area. As mentioned, observing CC was difficult because of the intense staining of the capsular fibers. In a few cases, lighter staining permitted tracing of their cell bodies and at least most of their dendrites but axons were not detected. Capsule cell dendrites were not observed extending beyond the capsule. The finding suggests that CC afferents are most likely from fibers in the capsule. Indeed, the capsule in SF has TH-ir and 5HT-ir fibers (Herbsman et al., 2000).

In several cases where two adjacent capsule cells were visible, their dendrites extended to nearly the level of the other capsule cell’s soma (Figure 12, also see Legend for Figure 9A). Connections between the overlapping dendrites are not apparent; however, the dendrites were not observed at the EM level. The findings suggest that CC might form “chains” of overlapping cells that extend the length of the capsule. Such a possible multicellular “system” might be topographically organized.

#### Area Dorsalis Telencephali, Pars Centralis-Dorsal Region

##### General Considerations

The general aspect of the SF Dcd, its connections, and known functions have been reviewed (Ito et al., 1982; Demski and Beaver, 2001; Demski, 2003, 2013; Xue et al., 2003a,b). This study added more detail concerning the cellular morphology of the area. The Dcd cells studied were from the central area in which cells were retrogradely filled from BDA implants in the tectum (Figure 13E; see above references). The Dcd cells stained in aggregates regardless of the technique (Figure 14B), and only a few individual cells could be traced or photographed (Figures 14Ab–d,C).

While Dcd cell bodies appear fairly dispersed (Figures 2, 3, 13E, 14bc), their expansive dendritic configurations form a massive entanglement (Figure 14B). The functional attributes of the overlap are unknown. Dcd projections to both the tectum (Demski and Beaver, 1997) and nPTh (Xue et al., 2003a) appear to be topographical. The transfer of such information from Dld1 (an at least rudimentary laminar structure) to Dcd (a seemingly unstructured nuclear mass) and then *via* Dcd axons back to the tectum (a highly laminar structure) is certainly an interesting area needing further study.

Area dorsalis telencephali, pars centralis-dorsal region cells have a soma with considerable Nissl material which is in accord with supporting a large dendritic tree and long axon (Peters et al., 1976). The longest dendrites illustrated in cells of Figure 14 are certainly the underestimates of total length since the thinnest parts of the processes were often cut off from the sections. Axons of Dcd cells may extend over 5–6 mm from Dcd to the tectum. The cells typically have 2–4 dendrites that branch from the soma and these have only a few main peripheral branches (Figures 14Ab–d,C). The sparse branching provides considerable dendritic shaft with spines receiving probable excitatory asymmetric synapses (Figures 13A,B). The small EM sampling does not permit comment on possible axosomatic connections.

The Dcd of SF is filled with scattered TH-ir fibers (Herbsman et al., 2000) and perineuronal satellites were associated with Dcd cells in one set of sections. The satellite cells mirror the appearance of microglia associated with cortical pyramidal cells. They may reflect a diseased condition (Peters et al., 1976).

##### Comparative Anatomical Considerations

In addition to SF, large central pallial cells are present in a variety of euteleosts (Sheldon, 1912; Bannister, 1973; Ito, 1973; Northcutt and Braford, 1980; Schroeder, 1980; Northcutt and Davis, 1983; Maler et al., 1991; Folgueira et al., 2004; Northcutt, 2006; Hagio et al., 2018, 2021). In some cases, the cells innervate the tectum as they do in SF (Ito and Kishida, 1977; Demski, 2013; Hagio et al., 2018), suggesting they are probably homologous within teleosts. Consistent with the situation in SF, TH-ir fibers are present in the Dc/Dcd in several fishes (Hornby et al., 1987; Kapsimali et al., 2000; Kaslin and Panula, 2001; Vetillard et al., 2002; Vacher et al., 2003; Wullimann and Mueller, 2004; O’Connell et al., 2011).

Including SF, a strong Dld input to Dcd cells has been demonstrated in various fishes (Murakami et al., 1983; Yamane et al., 1996; Demski, 2003, 2013; Xue et al., 2003b; Northcutt, 2006; Yamamoto and Ito, 2008). Groups of Dc cells appear to join with their overlying pallial regions and together form units (e.g., Dld1–Dcd in SF) with the Dc cells as an output component (Northcutt and Braford, 1980; Braford, 1995; Demski, 2013). NADPH-d activity and parvalbumin staining in developing zebrafish indicate a close developmental association of Dl and Dc such that they might be considered one structure. The area could be, at least in part, the dorsal pallium, a purported homolog of tetrapod isocortex (Mueller et al., 2011). As mentioned above, Dld1 alone probably does not have the structural diversity of a “cortex” but the combined developmental unit Dld1/Dcd might qualify as such. Indeed, Ito and Yamamoto (2009) described such areas as possible components of a “non-laminar isocortex.” Teleost homologies with isocortex are certainly not resolved (Ishikawa et al., 2007; Yamamoto et al., 2007, 2017; Mueller et al., 2011; Northcutt, 2011; Striedter and Northcutt, 2021).

### Tectal-Related Pallium-Tectal-Cerebellar Complex

**FIGURE 15:**
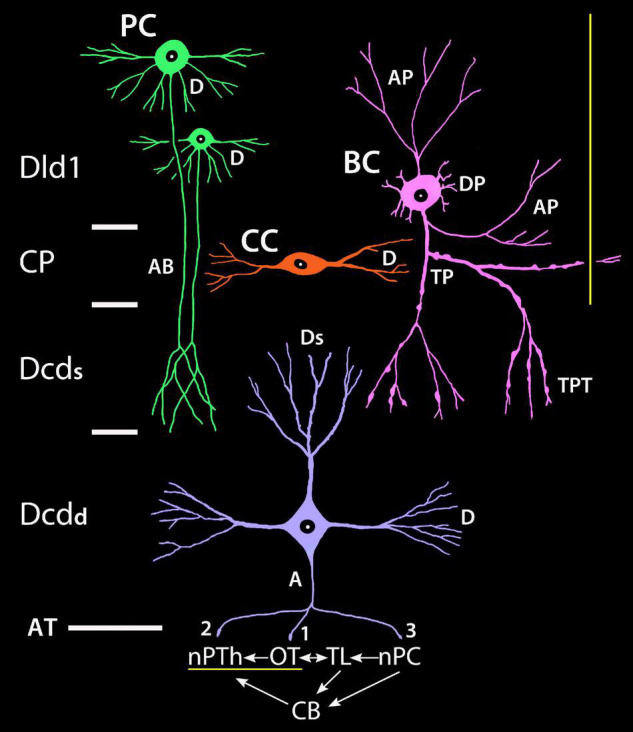
The present results on the cytoarchitecture of the squirrelfish “tectal-related pallium” (TRP) are summarized in this stylistic diagram with color-coded cellular components [PC; larger of two primary cells in green; BC; bridge cell in magenta; CC; capsule cell in orange; large cell of Dcd (area dorsalis telencephali, pars centralis-dorsal region) in light blue]. The TRP is divided into “horizontal zones” moving from the surface (top of figure) to deep, i.e., Dld1 [area dorsalis telencephali, pars lateralis-dorsal region (dorsal segment)]; CP (the fiber capsule separating Dld1 and Dcd); Dcds (the superficial area of Dcd); Dcdd (the deeper regions of Dcd). The zones are delineated on the left side by the shorter horizontal white bars. The longer white bar near the bottom of the figure indicates the area of Dcd cell axonal terminations (AT) with three structures extrinsic to the TRP: (1) optic tectum (OT); (2) nucleus prethalamicus (nPTh) and (3) nucleus paracommissuralis (nPC). The sensorimotor systems associated with these TRP-contacted structures are summarized by the white abbreviations separated by directional arrows. The systems are referenced in the “Introduction” and considered in more detail in the “Discussion” section. The major tectal input to the TRP is represented by: the yellow underlining of the OT to nPTh connection and the yellow vertical line in the upper left of the figure indicating the nPTh projection to the Dld1 zone with probable Dld1 afferent nPTh fibers running in the CP. Contrary to the actual condition, the cellular components of this figure area are depicted as non-overlapping in the zones they co-occupy. This “artificially expanded map” is used to “simplify” the “piece by piece” building of an integrated structural “model” of the region (see “Discussion” section). A, axon of Dcd cell; AB, PC axon bundles; AP, axon-like processes of the bridge cell; CB, cerebellum; D, dendrites; Ds, superficial dendrites of Dcd cell; DP, dendrite-like processes of the bridge cell; TL, torus longitudinalis; TP, thick processes of the bridge cells; TPT, terminations of the TP in Dcd. Structures are not drawn to scale.

The TRP of SF is likely a component in an organized group of circuits that regulate sensorimotor systems associated with vision, visuomotor control, and possibly spatial memory (Demski and Beaver, 2001; Demski, 2003, 2013; Xue et al., 2003a,b). Hypothetically, the complex integrates functions across the pallium, tectum, and cerebellum with the Dcd output connections (AT; numbered 1–3 in Figure 15) being a critical feature of several “loops” within the complex. Dcd connection 1 to multiple layers of the tectum is probably mostly related to vision. Connection 2 is to nPC, a direct relay to the cerebellum. Connection 3 is to the small-cell subdivision of nPTh. The cells probably modulate the output of nPTh large-cells which provide a major source of afferents to Dld1 (see yellow lines in Figure 15). Complex interconnections relate the TL (part of the tectum), the nPC, and the cerebellum. The latter has a projection to the plexiform subdivision of nPTh which also likely influences the large-cell output to Dld1. Thus, there are several levels of feedback probably mediated in part by nPTh internal connections.

The present results suggest another control component within “TRP-tectal-cerebellar complex,” i.e., the bridge cell. As described, the interconnections of bridge cells with Dld1 and Dcd could permit BC modulation of the functional relationship between principal cells and Dcd cells; e.g., the bridge cells are positioned to receive the same afferents as principal cells in Dld1 and the same PC output as the Dcd cells. It is likely that BC axons in Dld1 synapse on PC dendrites or perhaps on those of an intermediate cell that connects to principal cells (not identified but could be the smallest principal cells). Whichever the case, if the last link in the connection is inhibitory, the bridge cell “system” could be a short negative feedback regulator controlling the output of the TRP.

## Data Availability Statement

The raw data supporting the conclusions of this article will be made available by the authors, without undue reservation.

## Ethics Statement

The animal study was reviewed and approved by an Institutional Animal Care and Use Committee.

## Author Contributions

LD was the principal investigator on the NSF Grant funding this work as well as overseer of funds on the New College Leonard S. Florsheim Endowment to the New College Foundation, did the organization of the work and most of the interpretation of the results, wrote the manuscript with help from JB, in addition, regarding the Golgi studies, did most of the work other than sectioning and mounting of the tissues which was done by JB, and solely involved in work in Honduras while JB carried out most of the laboratory work in Sarasota, including the EM studies. JB helped in the production of the Golgi drawings and was in charge of animal care. Both authors contributed to the article and approved the submitted version.

## Conflict of Interest

The authors declare that the research was conducted in the absence of any commercial or financial relationships that could be construed as a potential conflict of interest.

## Publisher’s Note

All claims expressed in this article are solely those of the authors and do not necessarily represent those of their affiliated organizations, or those of the publisher, the editors and the reviewers. Any product that may be evaluated in this article, or claim that may be made by its manufacturer, is not guaranteed or endorsed by the publisher.

## Abbreviations

AB: axon bundle
AP: axon-like processes of bridge cell
Avt: area ventralis of the telencephalon
BA: beaded axons
BC: bridge cell
BDA: biotinylated dextran amine
BV: blood vessel
CB: cerebellum
CC: capsule cell
CP: fiber capsule between Dld and Dcd
Dc: area dorsalis telencephali, pars centralis
Dcd: area dorsalis telencephali, pars centralis-dorsal region
Dci: area dorsalis telencephali, pars centralis-intermediate region
Dcv: area dorsalis telencephali, pars centralis-ventral region
Dd: area dorsalis telencephali, pars dorsalis
Dl: area dorsalis telencephali, pars lateralis
Dld: area dorsalis telencephali, pars lateralis-dorsal region
Dld1: area dorsalis telencephali, pars lateralis-dorsal region (dorsal segment)
Dld2: area dorsalis telencephali, pars lateralis-dorsal region (ventral segment)
Dlv: area dorsalis telencephali, pars lateralis-ventral region
Dm: area dorsalis telencephali, pars medialis
Dm1: area dorsalis telencephali, pars medialis-periventricular nucleus
Dm2: area dorsalis telencephali, pars medialis-lateral nucleus
Dp: area dorsalis telencephali, pars posterior
DP: dendrite-like process of bridge cell
EM: electron microscopy
ER: endoplasmic reticulum
IL: inferior lobe
LFB: lateral forebrain bundle
M: meninges
MD: medulla
nPC: nucleus paracommissuralis
nPTh: nucleus prethalamicus
OB: olfactory bulb
OFN: olfactory nerve
ON: optic nerve
OT: optic tectum
PC: principal cell
SF: squirrelfish
T: telencephalon
TL: torus longitudinalis
TP: thick process of bridge cell
TPT: thick process terminations
TRP: tectal-related pallium
TS: thick section (ca. 1-μ m plastic section)
V: telencephalic ventricle.
